# Supplementary material for: Kinetics of plasma cfDNA predicts clinical response in non-small cell lung cancer patients
Source: Sci Rep. 2021 Apr 7;11:7633. doi: 10.1038/s41598-021-85797-z (PMC8027214; doi:10.1038/s41598-021-85797-z)
Supplement: Supplementary file 1 — Supplementary Information [file 41598_2021_85797_MOESM1_ESM.docx]

**Supplementary Material for**

**Kinetics of Plasma cfDNA Predicts Clinical Response in Non-Small Cell Lung Cancer** **Patients**

Xiaorong Zhou^1#^, Chenchen Li^1#^, Zhao Zhang^2#^, Daniel Y. Li^2#^, Jinwei Du^2^, Ping Ding^2^, Haiyan Meng^2^, Hui Xu^2^, Ronglei Li^3^, Effie Ho^2^, Aiguo Zhang^2^, Paul Okunieff^4^, Jianwei Lu^1*^, Michael Y. Sha^2*^

^1^ Jiangsu Cancer Hospital, Institute of Cancer Research, The affiliated Cancer Hospital of Nanjing Medical University, Nanjing, Jiangsu, China, 210009.

^2^ DiaCarta, Inc., 2600 Hilltop Drive, Richmond, California 94806 USA.

^3^ Genemile, Inc., Nanjing, Jiangsu, China.

^4^ Department of Radiation Oncology, University of Florida.

^#^These authors contributed equally.

*Correspondence should be addressed to J Lu ([lujw@medmail.com.cn](mailto:lujw@medmail.com.cn)) and M Sha ([msha@diacarta.com](mailto:msha@diacarta.com)).

**Supplementary Table 1**

**Comparisons of cfDNA levels and ratio by stage, therapy, and subtype**

|  |  | stratification | | | |  |
| --- | --- | --- | --- | --- | --- | --- |
| Stage |  | I | II | III | IV | P |
|  | n | 2 | 4 | 19 | 126 |  |
| cfDNA | baseline  (mean (SD)) | 20.47  (9.98) | 23.70  (21.39) | 19.38  (11.75) | 20.64  (15.40) | 0.96 |
|  | post-therapy (mean (SD)) | 21.58  (18.10) | 24.36  (9.90) | 26.14  (21.26) | 22.41  (14.43) | 0.79 |
|  | Ratio  (mean (SD)) | 0.95  (0.42) | 1.41  (0.80) | 1.48  (0.94) | 1.36  (0.92) | 0.86 |
| Therapy |  | CT-only | CT+VEGFIs | CT+TKIs | CT+ICIs | P |
|  | n | 2 | 53 | 15 | 126 |  |
| cfDNA | Baseline  (mean (SD)) | 20.12  (12.50) | 21.13  (15.46) | 19.74  (14.56) | 21.33  (17.52) | 0.97 |
|  | post-therapy (mean (SD)) | 24.33  (14.69) | 23.62  (14.34) | 16.11  (8.47) | 22.81  (18.19) | 0.32 |
|  | Ratio  (mean (SD)) | 1.49  (1.00) | 1.39  (0.81) | 1.04  (0.72) | 1.29  (0.97) | 0.38 |
| Subtype |  | LUAD | | LUSC | | P |
|  | n | 128 | | 26 | |  |
| cfDNA | Baseline  (mean (SD)) | 20.19  (14.15) | | 23.53  (18.87) | | 0.30 |
|  | post-therapy (mean (SD)) | 23.00  (14.16) | | 22.31  (19.76) | | 0.83 |
|  | Ratio  (mean (SD)) | 1.42  (0.93) | | 1.05  (0.71) | | 0.05 |

**Supplementary Table 2**

**Comparisons of CT plus VEGFIs between Ratio-High and Ratio-Low group**

|  | *Number* | |
| --- | --- | --- |
| Regimen | Ratio-High | Ratio-Low |
| BEV+PEM+CBP | 19 | 14 |
| BEV+PEM+CIS | 2 | 0 |
| BEV+PEM+NDP | 1 | 1 |
| BEV+PEM | 1 | 2 |
| Others | 7 | 6 |
| Sum | 30 | 23 |

BEV: Bevacizumab

PEM: Pemetrexed

CBP: Carboplatin

CIS: Cisplatin

NDP: Nedaplatin
